# Socioeconomic and Engagement Barriers to Cardiovascular Risk Awareness Among Cancer Survivors

**DOI:** 10.21203/rs.3.rs-8457803/v1

**Published:** 2026-01-21

**Authors:** Kristine J. Hahm, Dohyeong Kim, Carolyn Smith-Morris, Shelia Patterson, Marisel Ponton, Arthur S Hong, Lauren Taylor, Vlad Zaha

**Affiliations:** University of Texas at Dallas; University of Texas at Dallas; The University of Texas Southwestern Medical Center; Hoops Legends; The University of Texas Southwestern Medical Center; The University of Texas Southwestern Medical Center; The University of Texas Southwestern Medical Center; The University of Texas Southwestern Medical Center

**Keywords:** CVD risk awareness, Healthcare engagement, Cancer survivorship, Socioeconomic determinants, Health communication

## Abstract

**Purpose:**

To assess the prevalence of self-reported awareness of treatment-related CVD risk among cancer survivors and evaluate how socioeconomic, racial, clinical, and access-related factors shape awareness. A secondary objective was to examine non-linear and intersectional patterns.

**Methods:**

A cross-sectional survey of 614 cancer survivors was conducted between November 2023 and January 2025 to examine factors associated with awareness of treatment-related CVD risk. Respondents’ 10-year ASCVD risk was estimated using pooled cohort equations. Multivariable logistic regression estimated independent predictors, and decision tree analysis and interaction plots examined non-linear and intersectional patterns.

**Results:**

Respondents had a mean age of 54.3 years; 60% identified as Black/African American and 53% as female. Over half (54.2%) were classified as low ASCVD risk, while 16.2% were high risk. Awareness declined as clinical risk increased; with only 14% of high-risk survivors reporting awareness. Older age (β = 0.048, *p* < 0.001), lower income (β = −0.140, *p* = 0.010), and hypertension (β = −0.753, *p* = 0.005) were associated with non-awareness. Oncology engagement improved awareness, however, substantially more for White than Black/AA survivors (β =−1.178, *p* = 0.027). Decision tree showed that survivors with frequent engagement, sociodemographic factors impacted CVD risk awareness, whereas among those with fewer visits, structural access barriers were more influential.

**Conclusion:**

CVD risk awareness among cancer survivors remains low and patterned by socioeconomic and engagement-related factors.

**Implications for Cancer Survivors:**

Enhancing communication and engagement and addressing structural access barriers in survivorship care is essential to improve cardiovascular risk recognition and long-term health outcomes.

## Introduction

For an increasing proportion of cancer survivors, the risk of cardiovascular disease (CVD) related mortality exceeds that of cancer-specific mortality [1-5]. Nonetheless, many survivors continue to experience persistent challenges, including multiple comorbidities, adverse health behaviors, and long-term treatment-related effects [6]. While the cardiotoxic consequences of cancer therapies are well known among clinicians, many cancer survivors remain unaware of their elevated CVD risk and the importance of ongoing CVD risk management [7]. Survivors also emphasized the importance of oncology providers addressing cardiovascular health more explicitly as part of comprehensive survivorship care [8]. Effective communication about treatment-related cardiovascular risks is essential, as awareness itself has been shown to encourage healthier behaviors [9], which may, in turn, mitigate adverse cardiovascular outcomes associated with cancer therapies.

At the same time, substantial ethnic, racial, social, and geographic disparities persist in both cancer and CVD outcomes [10]. Social determinants of health, including socioeconomic status, education, employment, and neighborhood environment, significantly influence outcomes across both breast cancer and cardiovascular disease [11-13]. Awareness of CVD risk also appears to be disproportionately low among racial and ethnic minority populations [14].

This study investigated the socioeconomic and access-related factors that shape CVD risk awareness among cancer survivors. Specifically, we compared survivors’ self-reported awareness of treatment-related cardiovascular risk with their calculated clinical risk, as estimated by the 10-year ASCVD score, to evaluate concordance between perceived and objective risk. Furthermore, we employ a multi-method analytical approach, including chi-square tests, multivariable logistic regression, decision tree modeling, and interaction plots, to examine the complex intersections of socioeconomic and access-related determinants of awareness and to visualize how these factors collectively influence survivors’ recognition of cardiovascular risk.

## Methods

### Survey development

A cross-sectional survey was developed to assess self-reported cardiovascular disease (CVD) risk awareness among cancer survivors. The survey included items designed to capture knowledge of cancer treatment-related cardiovascular side effects as well as engagement in preventive health behaviors. In addition, respondents provided self-reported information on demographic characteristics, health behaviors and conditions, healthcare accessibility, frequency of care engagement, and residential zip code, allowing for examination of both individual- and contextual-level factors associated with CVD risk awareness.

### Survey recruitment

Participants were recruited through community-based cancer survivor organizations (50 Hoops and the Oak Cliff Bible Fellowship), clinical outreach, community postings, social media announcement, and direct emails to registered patients in the UTSW cardio-oncology clinic.

Eligible participants were adult cancer survivors (≥ 18 years) with a history of receiving any form of cancer treatment. A total of 614 cancer survivors provided informed consent and completed the survey. The survey was administered electronically to accommodate participants’ preferences and minimize accessibility barriers. Responses were anonymized, and participants were assigned unique identifiers to ensure confidentiality. Data was collected between November 2023 and January 2025.

### CVD Risk Awareness Measure

The primary outcome was self-reported CVD risk awareness, operationalized as respondents’ self-perceived knowledge of the potential cardiovascular effects of cancer treatments. Awareness was assessed with the question: *“Do you know if your cancer treatments impacted your heart health?”* Participants could respond with “Yes,” “No,” or “I don’t know.” For analytic clarity, responses were dichotomized: “Yes” (CVD risk aware), indicating that the respondent explicitly claimed awareness of treatment-related cardiovascular risks. “Non-Yes” (CVD risk unaware), a combined category encompassing both “No” and “I don’t know” responses, representing individuals who did not claim such awareness.

This dichotomization distinguished between those who actively acknowledge cardiovascular implications of their treatment and those who are either unaware or uncertain. It is important to note that this outcome represents self-reported or “claimed” awareness, which may not accurately reflect an individual’s objective understanding of CVD risk. As such, the findings primarily capture survivors’ perceptions rather than verified knowledge.

### Covariates and Confounders

Covariates included demographic, healthcare accessibility, clinical, and behavioral factors, selected based on scientific relevance and prior evidence [15, 16]. Demographic characteristics included age, race/ethnicity, gender, income level, and education level. Healthcare accessibility measures included: 1) travel time to primary care and oncology providers (in minutes), 2) reliability of transportation (never to always), 3) primary transportation mode (private vehicle vs. public/shared transportation). Health conditions and behaviors included history of smoking, diabetes, and hypertension, as well as whether respondents were receiving treatment for these conditions. Engagement with oncology care was operationalized as the frequency of follow-up visits to primary care and oncology providers (once a year to more than 4 times a month).

In addition, each participant’s 10-year atherosclerotic cardiovascular disease (ASCVD) risk was calculated using the pooled cohort equations, incorporating age, race/ethnicity, gender, smoking status, diabetes, hypertension, and treatment history [17]. To address age constraints inherent in ASCVD risk prediction models, individuals younger than 40 years were coded as age 40, while those older than 79 years were coded as 79 years. Individuals with missing race/ethnicity information were classified as White/Other for risk estimation. These standardized coding decisions allowed for the inclusion of all participants in risk modeling and maintained comparability across age and demographic groups.

Participants were also categorized into clinically relevant ASCVD risk strata based on the American College of Cardiology/American Heart Association (ACC/AHA) guidelines [18]. Since we relied on self-reported information on health and conditions, we had low confidence of obtaining accurate estimates of the several needed cholesterol values and blood pressure measurements. To overcome these issues, we used validated language from the National Health Interview Survey to obtain information on health condition elements (smoking history, diabetes status, hypertension status, hyperlipidemia status; age, race/ethnicity). We then estimated 10-year ASCVD risk assuming normal values of systolic blood pressure and cholesterol values. Imprecision of the estimated risk was further mitigated by relying on the risk categories (low-, intermediate-, and high-risk) instead of absolute percentage estimates. Categorizing survivors into these strata enabled us to contextualize their calculated cardiovascular risk relative to their self-reported awareness, providing a dual perspective on perceived vs. calculated risk. Although this was imperfect, this functionally improved the accuracy of self-report by patients, and limited survey question burden. However, we accepted that this would systematically underestimate estimated risk among our cohorts.

### Statistical Analyses

All analyses were conducted in a structured sequence to evaluate associations between participant characteristics and self-reported CVD risk awareness. Pearson’s chi-square tests were utilized to identify covariates significantly associated with self-reported awareness (Yes vs. Non-Yes) at an α level of 0.05. Significant variables were then included in multivariable logistic regression models to assess independent predictors of CVD risk awareness, adjusting for potential confounding factors, and estimate the adjusted odds of answering Non-Yes. The dependent variable was coded as *1 = Non-yes (Non-aware)* and *0 = Yes (Aware)*.

To assess whether relationships between predictors and awareness extended beyond linear and additive effects, a Classification and Regression Tree (CART) analysis was conducted. Decision trees are particularly well suited for detecting non-linearities and higher-order interactions among covariates [19], that may not be identifiable through regression-based approaches. To ensure model generalizability and prevent overfitting, cost-complexity pruning with k-fold cross-validation was applied. The final pruned model provided both a ranked hierarchy of predictor importance and a set of interpretable splitting rules illustrating how combinations of socioeconomic, behavioral, and access-related factors jointly structure survivors’ awareness.

Building on these results, interaction plots were used to visually examine key effect modifications, particularly across race, travel time, and care engagement, that were not fully captured by main effect alone. This integrated framework, using logistic regression, CART, and interaction plots, allowed for a more complete characterization of both independent effects and complex, intersecting mechanisms underlying disparities in CVD risk awareness.

Support for this research was provided by the Harold C. Simmons Comprehensive Cancer Center, Office of Community outreach, Engagement & Equity and the Cancer Center Support Grant (P30CA142543), and from the UT Southwestern Center for Translational Science Award (Community Engagement Planning Grant). This was reviewed and deemed accepted by the University of Texas Southwestern Medical Center Institutional Review Board (STU-2023-0839).

## Results

### Cohort characteristics

The final analytic sample included 614 cancer survivor respondents. The mean age of respondents was 54.3, 59.5% identified as Black/African American, and 37.6% were White. 53.3% were female. In terms of healthcare access, over one-third of respondents reported never had difficulty seeing a primary care provider, while 8.5% reported such challenges often, and 28% reported them rarely. Regarding transportation, 62.8% indicated having access to reliable transportation, and most used a private vehicle for medical appointments.

Health-related behaviors revealed that 37.3% of respondents had a history of smoking, 27.5% reported a diagnosis of diabetes, and 63.4% reported hypertension. Regarding oncology care engagement, approximately 16% reported visiting their oncology provider once a year, 45.8% several times per year, and 2.5% reported more than four times a month.

#### 10-year ASCVD Risk and Risk Awareness

Among the 574 respondents with complete data for 10-year ASCVD risk calculation, 54.2% were classified as low risk, 6.8% as borderline risk, 22.8% as intermediate risk, and 16.2% as high risk. Only 32% (n = 184) of these respondents were aware of the cardiovascular disease risk conferred by their cancer treatments.

A paradoxical pattern seemed to emerge; self-reported CVD risk awareness declined as estimated risk increased. Among individuals in the low-risk group, 44% reported knowing their risk, compared to only 26% in the borderline group, 18% in the intermediate group, and 14% in the high-risk group.

### Bivariate Associations with CVD Risk Awareness

Chi-square tests revealed that self-reported CVD risk awareness varied significantly across several demographic, access-related, and behavioral factors. Demographic factors included age (*p* < 0.001), race/ethnicity (*p* = 0.003), and income level (*p* = 0.0003). Access-related factors such as transportation time to primary care (*p* = 0.04) and oncology visits (*p* = 0.02), as well as difficulty accessing primary care, reliable transportation, and transportation method (all *p* < 0.001) were significant. Among behavioral and clinical variables, smoking history (*p* = 0.01), frequency of primary care and oncology visits (both *p* < 0.001) and ASCVD risk category (*p* < 0.001) were associated with self-reported awareness. All variables were carried forward into the multivariable logistic regression models.

### Multivariable Regression Results

The overall model demonstrated an acceptable fit (*AIC* = 453.07) and was based on 444 complete observations after accounting for missing data. After adjusting for demographic, socioeconomic, and health access variables, several predictors were significantly associated with self-reported non-awareness of CVD risk. ASCVD risk score was excluded from the analysis because it is a composite measure that incorporates age, race, and health conditions already included as covariates, which would have introduced redundancy.

Age was a strong positive predictor (β = 0.048, *p*< 0.001), indicating that older survivors were significantly more likely to lack awareness of their CVD risk. Income was inversely associated with non-awareness (β = −0.140, *p* = 0.010), suggesting that individuals with higher-income levels were more likely to recognize their cardiovascular risk. Likewise, respondents with hypertension had substantially lower odds of non-awareness (β = −0.753, p = 0.005), suggesting that those with existing clinical conditions may be more attuned to their cardiovascular health.

In contrast, travel time to care, both to primary care and to oncology doctor, was not statistically significant, implying that physical distance or transportation barriers alone did not explain differences in self-reported awareness. More importantly, the frequency of oncology visits appeared to play a critical role. While the main effect of frequent oncology visits was not significant in this model, the interaction between race and oncology visit frequency was significant (β = −1.178, p = 0.027). These findings indicate that proximity to healthcare facilities may not directly translate into effective risk communication. Moreover, the results underscore that the relationship between visit frequency and awareness differs by race, with White survivors who regularly visited their oncologist showing notably higher awareness levels compared to their non-White counterparts.

Overall, these findings suggest that awareness of treatment-related CVD risk is driven less by transportation or spatial access alone and more by the level and frequency of healthcare engagement and communication with providers, with variation across racial groups. Moreover, results from interaction terms indicated the presence of non-linear and effect-modifying relationships that may not be fully captured by traditional regression models. Accordingly, decision tree analysis was employed to more comprehensively characterize these complex and intersectional patterns.

### Decision Tree

Decision tree modeling, specifically the Classification and Regression Tree (CART) algorithm, was utilized to identify the influential predictors of self-reported cardiovascular disease (CVD) risk awareness among cancer survivors. Decision trees are non-parametric methods that iteratively split the data into increasingly homogenous subgroups, using measures such as impurity reduction to identify the variables and thresholds that best distinguish Yes from Non-Yes responses [19]. The CART algorithm further enhances this process by evaluating all possible splits to determine the most optimal partition [20]. This approach is particularly valuable for identifying complex, non-linear relationships between predictors while generating transparent and interpretable classification rules. Decision trees provide clear structures that clinicians and researchers can readily understand and validate [21]. Applied to survivorship care, where multiple demographic, behavioral, and access-related factors interact to influence CVD risk awareness, decision trees can reveal hierarchical patterns and subgroup differences, offering practical insights for addressing differences and informing patient-centered interventions.

A pruned classification tree was constructed excluding age and ASCVD risk to improve interpretability and reduce redundancy from composite measures. The final model identified frequency visit to primary care provider, race, gender, transportation reliability, travel time to care, education, and income as key predictors of self-reported CVD risk awareness.

Although primary care visit frequency was not statistically significant in the logistic regression model, the decision tree analysis identified it as the most influential predictor of awareness, emerging as the root node, the first and most decisive split in the model. This shows that while its linear effect may be obscured in the logistic framework, its predictive influence becomes clear when accounting for nonlinear relationships and interactions.

The first split occurred at frequency of primary care visits (≥ once a month), indicating that greater engagement with primary care was the first decisive factor predicting awareness. Among survivors with fewer primary care visits, awareness was predominantly shaped by structural access barriers, including reliable transportation and travel time to care, with additional stratification by income, education, and oncology visit frequency. In contrast, among survivors with more frequent primary care engagement, sociodemographic characteristics were more influential. Within this group, race emerged as the next major split, with White male respondents more likely to report awareness, followed by further differentiation by gender, perceived difficulty accessing care, and educational attainment.

Model performance indicated an overall accuracy of 81.1% (95% CI: 74.7–86.5), significantly higher than the no-information rate of 68.1% (p < 0.001). Specificity was high (88.8%), reflecting good classification of non-awareness, while sensitivity was moderate (64.4%). Predictive values were acceptable (PPV: 73.0%, NPV: 84.2%), and the ROC curve yielded an AUC of 0.795, reflecting good discriminative performance.

The variable importance ranked predictors by how much they reduced classification error across all tree splits. Consistent with the decision tree results, frequency of primary care visits was the most influential factor, followed by oncology visit frequency, perceived difficulty accessing care, and transportation reliability. These findings emphasize that healthcare engagement and access barriers, rather than demographic or behavioral characteristics, were the dominant predictors of awareness.

Overall, the pruned tree illustrates how awareness is shaped by intersections of socioeconomic and healthcare access-related factors rather than single predictors. Survivors with fewer primary care or oncology visits, greater difficulty accessing care, longer travel times, and unreliable transportation were more likely to be classified in the Non-Yes group, reflecting lower self-reported awareness. In contrast, those with more consistent healthcare engagement and fewer barriers were more likely to report awareness. These findings emphasize the combined influence of structural access constraints and healthcare utilization patterns on survivors’ recognition of cardiovascular risk.

### Engagement with Healthcare Providers

Because both the multivariable regression and the decision tree highlighted certain engagement and access-related factors as influential, the interaction plots were used to examine these relationships more closely. This closer inspection revealed racial differences in how these factors shape survivors’ CVD risk awareness.

Age emerged as the most prominent predictor of self-reported awareness in the regression analysis. The proportion of Non-Yes responses increased with age in both racial groups, reflecting lower awareness among older respondents. Black/AA respondents consistently demonstrated a higher proportion of non-awareness compared to White participants, with the gap most pronounced in younger age groups and attenuating modestly in later adulthood. These racial differences in awareness may reflect broader communication disparities in clinical encounters, as implicit racial bias among oncologists has been shown to reduce patient-centered communication and shorten interactions [22], potentially influencing how cardiovascular risk is conveyed to cancer survivors.

Travel time to either primary or oncology care was not statistically significant, indicating that distance alone did not meaningfully explain differences in survivors’ risk awareness. Black/AA survivors consistently reported higher non-awareness across all distance categories with minimal variation by travel time. For oncology care, again, Black/AA survivors showed higher non-awareness compared to White survivors.

Although visit frequency was not a significant main effect in the regression model, its interaction with race was significant, indicating that provider contact influences awareness differently across groups. The decision tree similarly highlighted visit frequency as a major contributor to awareness. Interaction plots showed that more frequent visits were generally associated with higher awareness, but the magnitude varied sharply by race. Among White survivors, non-awareness fell dramatically, from roughly 73% among those visiting only once a year to nearly 0% among those seeing primary care more than four times a month. In contrast, Black/AA survivors showed persistently high non-awareness across all visit frequencies, suggesting that increased contact with healthcare providers did not necessarily translate into better understanding of cardiovascular risk. This pattern also appeared for oncology visits, where awareness improved with more frequent visits for White survivors but remained relatively unchanged for Black/AA survivors.

These findings demonstrate that while more frequent clinical engagement enhances awareness for White survivors, communication quality and relational barriers may limit similar gains among Black/AA survivors, reinforcing persistent disparities despite comparable levels of healthcare use.

## Discussion

This study examined differences in CVD risk awareness among cancer survivors and found a persistent racial gap, with Black/AA survivors reporting substantially lower awareness than their White counterparts. Overall, lower awareness was associated with socioeconomic disadvantages and reduced healthcare engagement. In the multivariable logistic regression model, older age, individuals with lower income, and people without hypertension were significantly associated with higher odds of non-awareness. Although travel time was not statistically significant, the interaction between race and oncology visit frequency was, indicating that provider contact improved awareness for White—but not Black/AA—survivors.

Paradoxically, survivors in the highest 10-year ASCVD risk category were least likely to report awareness of their cardiovascular risk. Since ASCVD scores reflect demographic factors already linked to lower awareness (older age, Black/AA race), this pattern likely reflects the concentration of these groups in higher-risk tiers. This misalignment highlights a critical communication gap in survivorship care, where those most clinically vulnerable remain least informed [23]. Improving survivorship care requires more than clinical monitoring. It necessitates direct and accessible communication about cardiovascular vulnerability. Regular review of treatment protocols, paired with patient-focused education, may help reduce CVD risk after cancer therapies [24].

The decision tree revealed that survivors’ awareness was shaped more by socio-structural factors than clinical risk alone. After excluding age and ASCVD risk, variables such as visit frequency, perceived difficulty accessing care, travel time, race, income, gender, and education further distinguished levels of awareness, clustering those with compounded disadvantages in the Non-Yes group. Interaction plots further demonstrated that structural and engagement-related factors amplify these inequities. Across both primary and oncology care settings, Black/AA survivors maintained high non-awareness across all travel-time categories, while White survivors showed a non-linear pattern with awareness dipping at long distances. With increased visit frequency, awareness improved dramatically for White survivors but remained largely unchanged for Black/AA survivors, signaling persistent communication and engagement barriers.

These patterns suggest that simply improving access or visit frequency may be insufficient. These patterns highlight the pivotal role of patient-provider communication. When those at highest clinical risk do not receive or retain clear information about cardiovascular health, the benefits of healthcare access are diminished. Systemic inequities and implicit bias likely contribute to these communication gaps, reinforcing disparities even among survivors who are regularly engaged in care.

### Limitations

Several limitations should be acknowledged when interpreting these findings. First, the measure of CVD risk awareness relied on self-reported responses, which may be subject to recall bias, misperception, or social desirability. Survivors who responded Yes may be claiming awareness without necessarily possessing accurate knowledge of their risk factors, while those who responded Non-Yes may include individuals who are aware but uncertain about how to articulate their risk. This binary classification, although analytically useful, may therefore obscure more significant gradations of awareness.

Second, ASCVD risk estimates may systematically misclassify risk. While ASCVD risk scores are widely applied in clinical practice, their calibration has been questioned, particularly for populations outside the 40–79 age range or for non-White racial/ethnic groups [25-27]. It may also overestimate risk compared with newer models, yet it remains the standard tool for risk stratification in survivorship care and was therefore appropriate for our study population.

Third, the survey data did not distinguish whether reported income referred to individual or household earnings. This ambiguity limits interpretation of socioeconomic status and may underestimate or overestimate survivors’ true resource availability.

In addition, the relatively small sample size for decision tree modeling (N = 614) may limit the stability and generalizability of the classification results. Finally, the cross-sectional, self-reported survey design restricts causal inference and prevents assessment of changes in awareness over time. Future studies should use longitudinal data, validated awareness measures, and more precise socioeconomic indicators to better understand how structural inequities shape cardiovascular risk awareness among cancer survivors.

Despite regular engagement with healthcare providers, overall awareness of cardiovascular risk remained low and was shaped far more by socioeconomic and structural barriers than by clinical risk itself. Survivors facing multiple disadvantages, such as lower income, lower education, unreliable transportation, and greater difficulty accessing care, were disproportionately clustered in the Non-Yes group. A persistent racial gap also emerged, with Black/African American survivors exhibiting lower awareness even when healthcare utilization was comparable, suggesting that communication quality and systemic inequities may underlie these differences. These findings point to a systemic underemphasis on cardiovascular risk communication within survivorship care, reflecting evidence that many survivors receive little or no counseling on behavioral risk factor modification despite elevated CVD risk [28], particularly among Black/AA survivors who face compounded structural and communication-related barriers.

Improving survivorship care thus requires models that extend beyond individual behavior change. Healthcare providers must emphasize long-term chronic disease management and shared responsibility between primary care physicians and oncologists [1]. Key strategies include systematic monitoring of cardiovascular risk, strengthening patient-centered communication, and addressing logistical obstacles such as transportation to mitigate disparities. A comprehensive approach to cardio-oncology should also address structural determinants of health by reducing social and systemic barriers to care [11], enhancing education and resource allocation, and implementing targeted public health interventions [12], while deepening understanding of the mechanisms through which social determinants contribute to cardiovascular health disparities [13].

## Supplementary Material

This is a list of supplementary files associated with this preprint. Click to download.

• JouranlofCancerSurvivorshipSupplementaryfile.pdf

## Figures and Tables

**Figure 1:**
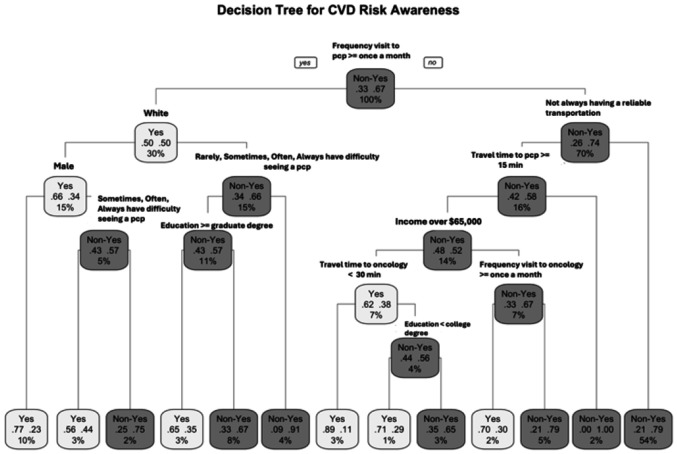
Pruned Decision Tree

**Figure 2:**
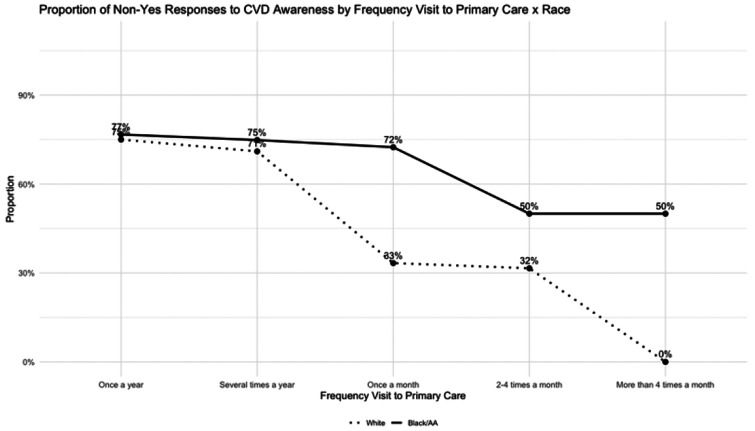
Proportion of Non-Awareness to CVD Risk by Frequency Visit to Primary Care x Race

**Figure 3:**
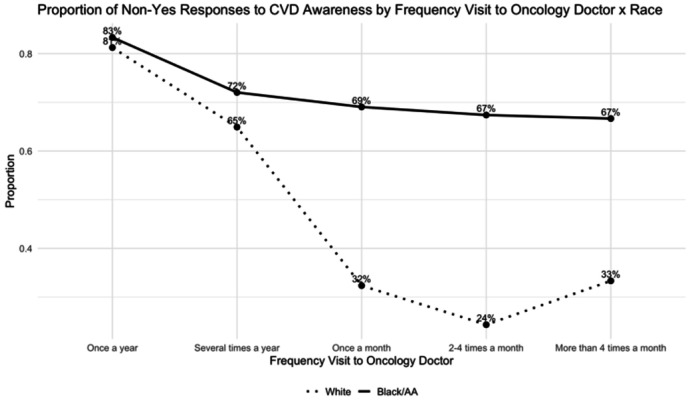
Proportion of Non-Awareness to CVD Risk by Frequency Visit to Oncology Doctor x Race

**Table 1.** Multivariable Regression Results

Variables	Coef	Std. Error	Z	Pr(>|Z|)
Logit *(Non-Yes vs Yes) N = 444 (due to missingness)*				
Constant	−1.321	0.996	−1.326	0.185
Age	0.048	0.010	4.628	<0.001***
Race (White)	−0.064	0.349	−0.183	0.855
Income	−0.140	0.055	−2.561	0.010*
Hypertension	−0.753	0.270	−2.794	0.005**
Travel Time to Primary Care (1 hour or more)	−1.164	0.895	−1.300	0.194
Travel Time to Oncology Doctor (1 hour or more)	0.534	0.642	0.831	0.406
Frequent Visit Primary Care (Over once a month)	0.155	0.380	0.407	0.684
Frequent Visit Oncology Doctor (Over once a month)	0.349	0.370	0.944	0.345
White X Frequent Visit Oncology Doctor (Over Once a month)	−1.178	0.534	−2.206	0.027*
	*AIC: 453.07*			

*** p <0.001;

** p <0.01;

* p <0.05

## Data Availability

The datasets generated during and/or analyzed during the current study are available from the corresponding author on reasonable request.

## Abbreviations

CVD: cardiovascular disease
ACVD: atherosclerotic cardiovascular disease
CART: classification and regression tree
PCP: primary care provider
CI: confidence interval
AUC: area under the curve
ROC: receiver operating characteristic

## References

[1] Choi K-H, Park SM, Lee K, Prevalence, Awareness, Control, and Treatment of Hypertension and Diabetes in Korean Cancer Survivors: A Cross-Sectional Analysis of the Fourth and Fifth Korea National Health and Nutrition Examination Surveys. Asian Pac J Cancer Prev 2013; 14: 7685–7692. 24460354 10.7314/apjcp.2013.14.12.7685

[2] Okwuosa TM, Anzevino S, Rao R. Cardiovascular disease in cancer survivors. Postgrad Med J 2017; 93: 82–90. 28123076 10.1136/postgradmedj-2016-134417

[3] Giza DE, Iliescu G, Hassan S, Cancer as a Risk Factor for Cardiovascular Disease. Curr Oncol Rep 2017; 19: 39. 28421481 10.1007/s11912-017-0601-x

[4] Strongman H, Gadd S, Matthews A, Medium and long-term risks of specific cardiovascular diseases in survivors of 20 adult cancers: a population-based cohort study using multiple linked UK electronic health records databases. The Lancet 2019; 394: 1041–1054. 10.1016/S0140-6736(19)31674-5 PMC6857444 31443926

[5] Wang Y, Wang Y, Han X, Cardio-Oncology: A Myriad of Relationships Between Cardiovascular Disease and Cancer. Front Cardiovasc Med 2022; 9: 727487. 35369296 10.3389/fcvm.2022.727487 PMC8968416

[6] Han X, Robinson LA, Jensen RE, Factors Associated With Health-Related Quality of Life Among Cancer Survivors in the United States. JNCI Cancer Spectr 2021; 5: pkaa123. 33615136 10.1093/jncics/pkaa123 PMC7883550

[7] Knowles R, Kemp E, Miller M, “There could be something going wrong and I wouldn’t even know”: a qualitative study of perceptions of people with cancer about cardiovascular disease (CVD) risk and its management. J Cancer Surviv 2025; 19: 319–325. 37775614 10.1007/s11764-023-01468-0 PMC11813814

[8] Weaver KE, Dressler EV, Smith S, Cardiovascular health assessment in routine cancer follow-up in community settings: survivor risk awareness and perspectives. BMC Cancer 2024; 24: 158. 38297229 10.1186/s12885-024-11912-8 PMC10829276

[9] Alzaman N, Wartak SA, Friderici J, Effect of Patients’ Awareness of CVD Risk Factors on Health-Related Behaviors: South Med J 2013; 106: 606–609. 24192590 10.1097/SMJ.0000000000000013

[10] Taylor LL, Hong AS, Hahm K, Health Literacy, Individual and Community Engagement, and Cardiovascular Risks and Disparities. JACC CardioOncology 2024; 6: 363–380. 38983375 10.1016/j.jaccao.2024.03.010 PMC11229558

[11] Obeidat O, Charles KR, Akhter N, Social Risk Factors That Increase Cardiovascular and Breast Cancer Risk. Curr Cardiol Rep 2023; 25: 1269–1280. 37801282 10.1007/s11886-023-01957-9 PMC10651549

[12] Ganatra S, Dani SS, Kumar A, Impact of Social Vulnerability on Comorbid Cancer and Cardiovascular Disease Mortality in the United States. JACC CardioOncology 2022; 4: 326–337. 36213357 10.1016/j.jaccao.2022.06.005 PMC9537091

[13] McFadden E, Luben R, Wareham N, Occupational social class, risk factors and cardiovascular disease incidence in men and women: a prospective study in the European Prospective Investigation of Cancer and Nutrition in Norfolk (EPIC-Norfolk) cohort. Eur J Epidemiol 2008; 23: 449–458. 18509727 10.1007/s10654-008-9262-2

[14] Mosca L, Mochari-Greenberger H, Dolor RJ, Twelve-Year Follow-Up of American Women’s Awareness of Cardiovascular Disease Risk and Barriers to Heart Health. Circ Cardiovasc Qual Outcomes 2010; 3: 120–127. 20147489 10.1161/CIRCOUTCOMES.109.915538 PMC2956447

[15] Underwood J. M., Townsend J. S., Stewart S. L., Buchannan N., Ekwueme D. U., Hawkins N. A., … & Fairley T. L. (2012). Surveillance of demographic characteristics and health behaviors among adult cancer survivors—Behavioral Risk Factor Surveillance System, United States, 2009. MMWR Surveill Summ, 61(1), 1–23. 22258477

[16] Weaver KE, Foraker RE, Alfano CM, Cardiovascular risk factors among long-term survivors of breast, prostate, colorectal, and gynecologic cancers: a gap in survivorship care? J Cancer Surviv 2013; 7: 253–261. 23417882 10.1007/s11764-013-0267-9 PMC3756807

[17] Goff DC, Lloyd-Jones DM, Bennett G, 2013 ACC/AHA Guideline on the Assessment of Cardiovascular Risk. J Am Coll Cardiol 2014; 63: 2935–2959. 24239921 10.1016/j.jacc.2013.11.005 PMC4700825

[18] Arnett DK, Blumenthal RS, Albert MA, 2019 ACC/AHA Guideline on the Primary Prevention of Cardiovascular Disease. J Am Coll Cardiol 2019; 74: e177–e232. 30894318 10.1016/j.jacc.2019.03.010 PMC7685565

[19] Song Y, Lu Y. Decision tree methods: applications for classification and prediction. Shanghai Arch Psychiatry; 27. 10.11919/j.issn.1002-0829.215044 PMC4466856 26120265

[20] Mohammadzadeh F, Noorkojuri H, Pourhoseingholi MA, Predicting the probability of mortality of gastric cancer patients using decision tree. Ir J Med Sci 1971 - 2015; 184: 277–284. 10.1007/s11845-014-1100-9 24626962

[21] Cruz JA, Wishart DS. Applications of Machine Learning in Cancer Prediction and Prognosis. Cancer Inform. PMC2675494 19458758

[22] Penner LA, Dovidio JF, Gonzalez R, The Effects of Oncologist Implicit Racial Bias in Racially Discordant Oncology Interactions. J Clin Oncol 2016; 34: 2874–2880. 27325865 10.1200/JCO.2015.66.3658 PMC5012663

[23] Hart J. T. The inverse care law. The lancet 1971; 297: 405–412. 10.1016/s0140-6736(71)92410-x 4100731

[24] Kapoor A, Prakash V, Sekhar M, Monitoring risk factors of cardiovascular disease in cancer survivors. Clin Med 2017; 17: 293–297. 10.7861/clinmedicine.17-4-293 PMC6297664 28765402

[25] Rana JS, Tabada GH, Solomon MD, Accuracy of the Atherosclerotic Cardiovascular Risk Equation in a Large Contemporary, Multiethnic Population. J Am Coll Cardiol 2016; 67: 2118–2130. 27151343 10.1016/j.jacc.2016.02.055 PMC5097466

[26] DeFilippis AP, Young R, McEvoy JW, Risk score overestimation: the impact of individual cardiovascular risk factors and preventive therapies on the performance of the American Heart Association-American College of Cardiology-Atherosclerotic Cardiovascular Disease risk score in a modern multi-ethnic cohort. Eur Heart J 2016; ehw301. 10.1093/eurheartj/ehw301 PMC5837662 27436865

[27] Mathew RO, Khan SS, Tuttle KR, Performance of the American Heart Association’s PREVENT risk score for cardiovascular risk prediction in a multiethnic population. Nat Med 2025; 31: 2655–2662. 40615687 10.1038/s41591-025-03789-2

[28] Christian AH, O’Malley D, Barac A, Cardiovascular risk and communication among early stage breast cancer survivors. Patient Educ Couns 2017; 100: 1360–1366. 28215826 10.1016/j.pec.2017.02.010 PMC5568653

